# Between privilege and exclusion: Orthodox church singers coping with the Covid-19 lockdown

**DOI:** 10.1177/00846724231158409

**Published:** 2023-03-04

**Authors:** Maria Takala-Roszczenko

**Affiliations:** University of Eastern Finland, Finland

**Keywords:** Coping, music, religion, wellbeing

## Abstract

The outbreak of the Covid-19 pandemic restricted public worship in many religious communities. This article explores how the amateur singers in Eastern Orthodox Christian church choirs coped with the 2-month liturgical lockdown in Finland during the spring of 2020. During the lockdown, only a limited number of singers were allowed to perform in worship, which was live streamed on social media. Based on a mixed-methods online survey, the article focuses on the psychological impact of the lockdown on individual church singers; their views of the spiritual, physical, mental and communal dimensions of singing in worship; and the methods of coping that their responses reveal. The analysis uses the religious coping theory developed by Kenneth Pargament and his colleagues. The results highlight how the church singers sought to maintain control of their lives in times of great uncertainty by focusing on individual religious activities and participating in live streamed worship. A new kind of autonomy was generated by the accessibility of worship on mobile devices, not confined to time or place. The results also reveal the negative effects of the lockdown on the social dynamics of the choir, the sense of being excluded from the community, as well as the guilt for having the privilege to sing in worship.

## Introduction

Finland is one of the European countries that sailed through the first wave of the Covid-19 pandemic in the spring of 2020 with relatively little damage (Safety Investigation Authority, 2021). One reason for this was the rapid decision of the Finnish government to restrict travel and public assembly. On 18 March 2020, Finland went into a 2-month lockdown, closing national borders, banning gatherings of more than 10 people and moving schools and universities into distance learning. The government also advised that churches and other religious communities restrict their public activities.

The Orthodox Church of Finland, which is one of the two state-supported churches with its c. 58,000 members (c. 1.05% of the population of Finland), alongside the Evangelical Lutheran Church, responded promptly and recommended that, from 18 March on, worship continue behind closed doors in the presence of maximum 10 people, meaning the officiating priest, the cantor, performing music and the necessary assistants. The congregants were to attend worship digitally.^1^ In some parishes, it was possible to participate in the Holy Communion immediately after the Divine Liturgy by entering the church in small groups. Besides this, the church buildings were not, as a rule, open for private prayer.

In times of crisis, people often turn to religion for solace (Pargament et al., 2013). In Orthodox Christianity, practicing religion is characteristically a social act (Luehrmann, 2018), primarily associated with communal liturgy in the physical church space. Orthodox liturgy is distinctly corporeal and rich in sensory experience (Gschwandtner, 2019). When congregational worship was transformed, practically overnight, into digital form, the congregants were deprived not only of the traditional venues and structures of worship but also of its profoundly embodied experience and the liturgical community.

The psychological impact of the March–May 2020 lockdown of liturgy on Orthodox believers has not yet been a focus of research. In their empirical study on how Finnish Orthodox experienced live streamed worship during the pandemic, Metso and Ahonen (2022) note how the restrictions on liturgical participation instigated feelings of disappointment and estrangement among some of their respondents, while for others, attending online worship could at least sustain the feeling of connection with the congregation. They point out that the pandemic challenged individual believers’ relationship with faith and the church, which is likely to have profound effects on their experience of the liturgical community in the future.

The disintegrative effects of the pandemic become visible on many different levels of congregational life. This article focuses on a particular group of Finnish Orthodox congregants, the amateur church singers and their coping with the lockdown of liturgy during the spring of 2020. Church singers are indispensable for a traditional Finnish Orthodox congregation where liturgical singing is purely vocal and predominantly performed by a mixed choir, rather than the whole congregation. Their role actualizes in the sacred space and context, that is, the liturgy. For many, singing is the main – if not the sole – way of participating in worship.

From the outset of the pandemic, singing became highlighted as a high-risk activity due to the increased amount of aerosols it releases (Charlotte, 2020; Gregson et al., 2021; Vance et al., 2021). Group singing was generally discouraged, and religious communities throughout the world faced the question how to practice music in worship without compromising the health of its performers (Freeman, 2021; Galadza, 2020; Mora & Martínez, 2022; Prassl, 2020). In the Orthodox Church of Finland, each parish was at liberty to organize their liturgical music within the restrictions recommended by the church. Since worship continued within church buildings, it never went completely virtual; in other words, it was transmitted and not hosted fully online. The cantor continued to sing in person, either alone, or assisted by one to three selected singers. Yet parishes adopted different strategies in implementing the restrictions, which meant that the singers could be seen as unequally positioned against each other since some were able to sing in church, while others were not. These differences became visible and audible in the live streamed liturgical services, enabling comparison between parishes and local churches.

In their study on the reactions to restricting choral singing in Sweden and Norway during the Covid-19 spring of 2020, Theorell et al. ([2023], p. 146. e19) correctly pointed out that while the benefits of choir singing have been widely examined, ‘very few have examined the effects when someone loses access to it and stops singing’. They surveyed the perceived loss among professional and amateur singers of classical, gospel, jazz, pop, folk music and other genres, with a specific focus on aspects such as *social bonding, something to look forward, possibility to experience flow, aesthetic experiences, physical exercise, breathing training* and *voice training*. Based on a substantial sample (n = 3163 in Sweden, n = 1991 in Norway), they concluded that the social aspect of singing was the element most missed during the lockdown.

With a considerably smaller sample (n = 120) and the focus exclusively on the amateur singers of church music within an Eastern Orthodox minority, my study is expected to highlight somewhat different aspects of choral singing, for example, in relation to spirituality. Yet given the communal character of Orthodox liturgy, it is quite likely that the perceived social dimensions of singing correspond to the findings of Theorell et al. (2023). Based on a survey conducted in June 2020, at the end of the 2-month lockdown in Finland, this study explores the psychological effects of the disruption of choral activities as reported by the church singers themselves. Drawing from the religious coping theory developed by Kenneth I. Pargament (1997), the lockdown of liturgy is seen as a critical life event that challenged the cohesion of the choral community and triggered different processes of coping among individual singers. The article addresses the following questions: How do individual singers generally perceive singing in worship? How did the lockdown impact their experience? What coping processes may be discerned in their reactions?

## Methods and theory

### Study design and implementation

The survey was designed by the author as a mixed-methods study, collecting both quantitative and qualitative data on the perceived benefits of singing in worship and on the church singers’ reactions to the lockdown. It was distributed in the form of an electronic questionnaire by e-mailing the cantors in the 21 parishes that comprised the Orthodox Church of Finland in 2020. The link was also posted on social media, using the Facebook page of the Finnish Orthodox Church (Ortodoksit Suomessa). Participation was voluntary and responding anonymous. The participants were informed about data privacy and asked to give their consent for research prior to responding. The questionnaire was open from 1 to 30 June 2020.

The data were then exported from Eduix E-lomake software to Excel. Quantitative data were coded and tabulated, and the open-ended responses were analysed qualitatively to find common patterns and themes. In the process of categorization, the measure of religious coping developed by Kenneth L. Pargament, known as the RCOPE (Pargament et al., 2000), was used for a theoretical framework that helped in discerning relevant points in the data. Subsequently, the qualitative content analysis of the data was conducted using the RCOPE tool. The results were grouped into three categories: embodied, spiritual, and communal experiences related to (not) singing during the lockdown of 2020.

### Participants

The data consist of responses from 120 church singers whose age range is as follows: under 25 (15; 12%), 25–40 (13; 11%), 40–55 (29; 24%), 55–65 (38; 32%), over 65 (25; 21%) years. The response rate may be considered adequate. Although there are no recent statistics available of the number of Orthodox church choirs, based on the count of parishes and contacts with choir leaders, it may be estimated that in 2020, their total was approximately 50, their sizes ranging roughly between 4 and 25 singers. Thus, the data (N = 120) average out at about 2.4 respondents per choir. Since the likelihood of compromising the anonymity of individual respondents from the smallest choirs was considerable, the participants were not asked to define their gender or local parish. Their geographical distribution all over Finland can be confirmed from responses concerning their diocese (51% respondents from the Archdiocese of Helsinki in southern Finland; 42% from the eastern Diocese of Kuopio and Karelia; 7% from the northern Diocese of Oulu).

### Quantitative survey

The questionnaire gathered information about the participants’ age and location (diocese), their frequency of singing at church before the pandemic and whether and how often they had sung in worship during the lockdown. Furthermore, they were asked about the organization of music in their parish, that is, had the cantor sung alone or with a group of choir singers, and had the choir continued to function during the lockdown. They were also asked whether they had attended live streamed services.

Next, they were asked to complete two scales. The first (five-item) scale explored the participants’ feelings about singing in worship in normal times. The scale was modelled on Moss et al. (2018) who grouped the benefits of choral singing into four main categories (spiritual, physical, emotional and social), with some modification: the social aspect was divided into ‘connection with the choir members’ and ‘connection with the congregation or the church’, and the emotional category was replaced with ‘mental wellbeing’.

The second (six-item) scale concerned the participants’ reactions to (not) singing in worship during the lockdown. The participant was expected to choose the relevant scale based on whether they had sung in worship. Although not explicitly named, the items of the second scale correlated to a certain degree with the four categories of the first scale, exploring the spiritual benefits (singing as *spiritually strengthening* vs lacking the chance to sing as *spiritually challenging*) and the physical benefits (*physically strengthening* vs *physically challenging*) of singing, as well as the mental aspects (singing as *stressful* or *laborious* vs lacking the chance to sing as *unfair* or *causing anxiety*), and the social, communal function (singing as *a responsibility* and *a privilege* vs lacking the chance to sing as *relieving* or causing feelings of *exclusion*). The two sets of items were not designed to constitute equal pairs on a positive–negative axis (e.g. *fair–unfair*); rather, the author determined these categories on the basis of reactions encountered on social media (comments by choir singers on live streamed services, etc.). The responses were given on a 5-point Likert-type scale (5 = *strongly agree*, 4 = *somewhat agree*, 3 = *cannot say*, 2 = *somewhat disagree*, 1 = *strongly disagree*). It was possible to leave questions unanswered.

### Qualitative survey

Participants were encouraged to complement their responses on the scales by elaborating on each item in their own words. The scales thus functioned as leading questions for the respondents to reflect on their feelings on singing and their reactions to the lockdown, to provide qualitative data. After this, they were asked to answer two open-ended questions: first, whether and what precisely they had most missed about singing in worship during the lockdown, and second, which moments they would have liked to share as choir singers during this time. The questionnaire ended with space for participants to write free responses.

### Religious coping theory

This theoretical framework for this article is the religious coping theory developed by Kenneth I. Pargament. The role of religion and spirituality in coping with critical life events has received increasing attention in the past decades. Especially since the publication of Kenneth I. Pargament’s (1997)*The Psychology of Religion and Coping*, and the subsequent validation of the RCOPE, a theory-based tool for measuring religious coping (Pargament et al., 2000), and its concise version, the Brief RCOPE (Pargament et al., 2011), the multifaceted involvement of religion in coping with crisis has been recognized and surveyed in a variety of denominational, demographic, ethnic and cultural contexts. Since its early focus on Western Christian populations, RCOPE-based research has become more diverse, adapted and validated, for example, in the context of Judaism, Islam (e.g. Abu-Raiya et al., 2020; Mohammadzadeh & Najafi, 2016) and Eastern Christianity with a Greek Orthodox focus group (Paika et al., 2017).

According to Pargament et al. (2013), the involvement of religion in coping should be explored with a wide spectrum of functions by focusing not only on

*how much* religion is involved in coping, but also *how* religion is involved in coping; specifically the *who* (e.g. clergy, congregation members, God), the *what* (e.g. prayer, Bible reading, ritual), the *when* (e.g. acute stressors, chronic stressors), the *where* (e.g. congregation, privately), and the *why* (e.g. to find meaning, to gain control) of coping. (pp. 562–563)

To survey how exactly individuals utilize religion in dealing with life stressors, the RCOPE measures 21 types of religious coping that derive from the functions of *meaning, control, comfort, intimacy, life transformation* and the *search for the sacred or spirituality itself*. Coping methods are assessed in 105 statements such as ‘Saw my situation as part of God’s plan’, ‘Felt my church seemed to be rejecting or ignoring me’ or ‘Offered spiritual support to family or friends’, which specify different cognitive, emotional and relational actions of coping (Pargament et al., 2011, p. 54).

Importantly, religious coping may be positive or negative. Positive coping reflects ‘a secure relationship with God, a belief that there is a greater meaning to be found, and a sense of spiritual connectedness with others’ (Abu-Raiya et al., 2020, p. 203), whereas negative coping is characterized by tension, discontent, even conflict. Pargament et al. (2011) emphasize that the efficacy of coping methods is determined by several factors and thus a seemingly negative coping method may lead to long-term growth and wellbeing, and vice versa.

This article tests the religious coping theory on data that were collected using mixed-methods. Unlike the majority of studies that have employed the RCOPE (e.g. Abu-Raiya et al., 2020; Paika et al., 2017; Talik, 2013), this study does not comprise a factor analysis, but uses the theory rather as an analytical tool, qualitatively, for categorizing and analysing the data.

## Results and discussion

### Quantitative results

In general, the results gathered with quantitative methods constitute an image of the Finnish Orthodox church singers as highly committed and motivated members of their congregation. Upon asking how frequently they had normally sung at church before the pandemic, 30% (36) of all respondents stated they had sung approximately once a week, and 27% (32) more than once a week. In a typical Finnish Orthodox parish, divine services take place every weekend, at the minimum on Saturday evening and on Sunday morning. Most parishes offer one or two weekday services, while there are approximately four to five parishes where attending church almost every day is possible. Thus, over half of the respondents had participated as singers in worship very committedly, every week. Participating twice a month was marked by 23% (28) of the respondents, and 13% (16) had sung approximately once a month. Only eight respondents (7%) had sung less frequently than once a month.

The lockdown disrupted the regular pace of singing. During the 2 months, 43% (52) of the participants had been able to continue singing at church, on average four to five times each. Consequently, 57% (68) had not sung in physical worship. Most of the participants had attended online worship during the lockdown; only 12% (14) had not watched the live streams at all.

Before exploring the participants’ reactions to the lockdown, they were asked to evaluate how beneficial they perceived singing in worship under normal conditions (Table 1).

**Table 1.** Perceived benefits of singing in worship in normal times.

Item	Mean	Standard deviation
A. Spiritual wellbeing (prayer, peace of soul, connection with God)	3.72	3.2
B. Physical wellbeing (energy, elation)	3.61	3.3
C. Mental wellbeing (good mood, balance, stabilization)	3.75	3.1
D. Connection with the choir members	3.69	3.3
E. Connection with the congregation or the church	3.79	3.2

Responses N = 119, except for point B (N = 117). The responses were coded as 4 for SA (‘Strongly agree’), 3 for A (‘Somewhat agree’), 2 for D (‘Somewhat disagree’) and 1 for SD (‘Strongly disagree’). The data for ‘Cannot say’ were excluded from the mean and the standard deviation.

As can be seen, singing was clearly recognized as a medium for connecting with the congregation or the church, which may have both social and spiritual implications. Some respondents complemented their answer by describing singing as *participation*, or as their *ministry* through which they served the *community*. This gave them a sense of *being useful*. It is also possible to note that the participants emphasized the mental and the spiritual benefits of singing in worship. Many highlighted the meaningfulness of singing with appreciative words such as *pacification, peace, balance, joy* and *good feeling*, even *success. Prayer* was also commonly mentioned. It is of course possible that the questions encouraged the respondents to recognize such feelings in themselves. Moreover, looking back to the pre-pandemic time may have flavoured the answers with certain nostalgia.

To explore the reactions to the disruption of regular participation in liturgical singing, the participants were asked to evaluate how access or the lack of it had impacted their experience (Tables 2 and 3). As noted earlier, the participants were expected to choose the relevant scale based on whether they had been able to sing in worship during the lockdown. However, the responses were not systematically distributed: compare the total number of responses in Tables 2 and 3 with the above statistics (52 participants had sung while 68 had not).

**Table 2.** Reactions to singing in worship during the lockdown.

Item	N	SA	A	D	SD	Mean	Standard deviation
Spiritually strengthening	55	35	15	5	0	3.55	3.1
Physically strengthening	39	17	17	5	0	3.31	2.8
Stressful	44	8	21	11	4	2.75	2.4
Laborious	46	2	2	13	29	1.5	1.2
Responsibility	47	27	19	1	0	3.55	3.1
Privilege	49	39	7	1	2	3.69	3.2

The responses were coded as 4 for SA (‘Strongly agree’), 3 for A (‘Somewhat agree’), 2 for D (‘Somewhat disagree’) and 1 for SD (‘Strongly disagree’). The data for ‘Cannot say’ were excluded from the mean and the standard deviation.

**Table 3.** Reactions to not being able to sing in worship during the lockdown.

Item	N	SA	A	D	SD	Mean	Standard deviation
Spiritually challenging	72	24	26	10	3	2.74	2.5
Physically challenging	70	6	15	22	18	1.68	1.8
Unfair	69	6	18	12	19	1.75	1.7
Causing anxiety	73	11	20	19	14	2.14	2.0
Relieving	70	2	14	18	22	1.54	1.4
Exclusive	83	15	27	9	10	2.04	2.1

The responses were coded as 4 for SA (‘Strongly agree’), 3 for A (‘Somewhat agree’), 2 for D (‘Somewhat disagree’) and 1 for SD (‘Strongly disagree’). The data for ‘Cannot say’ were excluded from the mean and the standard deviation.

The results show that the respondents valued the chance to take part in worship as a privilege, which is understandable since not everyone was admitted to physical worship. Many saw it also as a responsibility, which may be explained by the fact that singing in worship usually meant performing for live streams: it was not a task to be performed carelessly. More than half admitted that it was a stressful task, possibly due to its public nature, yet it was not considered too laborious. The respondents continued to recognize the spiritual benefits of singing at church, and for many, the experience was emotionally strengthening. Interestingly, the spiritual aspect also gathered responses from those who had not had access to physical worship.

Considering that singing in worship was felt to be spiritually beneficial, it is hardly surprising that more than half of the respondents saw its loss was seen as spiritually challenging. The situation caused considerable anxiety. A little more than half stated that being left out of physical worship instigated a feeling of exclusion. Interestingly, the strongly agreeing respondents represented all age groups, from under 25 to over 65 years, which indicates the feeling was not dependent on how long one had sung in the choir. Yet much fewer considered the situation as unfair: it is quite clear that the restrictions were understood as protecting people. Some explained that they were ‘all in the same boat’ – one just had to stick it out. The results show that although the lockdown relaxed busy schedules, only a few regarded this state of things as relieving.

What specifically draws the attention in Table 3 is the fact that the number of respondents exceeds the number of those who lacked access to worship (68) in all items. One explanation could be that the experience of not having access to physical worship during the lockdown was widely shared among church singers, as few had the chance to participate in every service.

### Qualitative results: analysis of religious coping

Pargament et al. (2013) argue that in crisis situations, an individual’s coping relies on a general orienting system, a ‘general disposition that involves beliefs, feelings, relationships, and practices embedded in religious, personality, and social domains’ (p. 566). For a committed church singer, attending church would most likely have provided a coping strategy in other kinds of conditions, yet during the lockdown, the solace of the church remained closed to many. The pandemic threatened to estrange the church singers from their ministry, which most of the respondents normally saw as enhancing their mental, physical and spiritual wellbeing, as well as having significant social functions. The analysis of the qualitative results of this study from the perspective of religious coping reveals the impact of the lockdown on the physical, spiritual and communal experience of singing, as described by the church singers themselves.

#### The embodied experience of singing

The lockdown was characteristically physical in its effect. By restricting physical assembly and closing public spaces such as churches, it was hoped to keep the risks of infection under control. From the perspective of Orthodox liturgy, the restrictions stripped the worship, first, of its body of worshippers, and with them, the collective experience of the ‘profoundly “embodied” expression of the Christian faith: [. . .] gestures, images, sounds, tastes, and scents that, together with the words, express the truths of the faith’ (Coleman, 2014, p. 11). Divine services may have continued in the physical space of the church, yet the experience was very different for those who officiated, as it was for those who attended the service online, as tangible participation became virtual.

The change was particularly drastic for those whose experience of worship was based on vocal participation. In pre-pandemic conditions, the embodied physicality of singing was clearly recognized as a source of wellbeing (see Table 1). The physical benefits of singing were described in this context as energizing, balancing or purifying: ‘It revitalizes the mind, soul, and body – renews/purifies the whole person’, described one respondent (R94). Others also emphasized the calming and balancing effect of singing: ‘I gradually become calm, let my shoulders fall, I breathe, concentrate, receive more energy than submit, I gain better balance’ (R85). ‘A balanced feeling, wellbeing, calming after stressful work’ (R24).

These notions are consistent with research. Singing is known to enhance an individual’s wellbeing in various ways, from parent–infant bonding to preventing cognitive decline, improving quality of life and coping with different diseases (Moss et al., 2018). The experienced benefits of singing have much to do with the brain hormone oxytocin which has been shown to mediate social behaviour and regulate stress and anxiety (Keeler et al., 2015). In an empirical study on professional and amateur singers during singing lessons in Sweden, Grape et al. (2002 see also Norton, 2016) noted a significant increase of oxytocin that singing effected. All participants reported energy and relaxation after singing, and moreover, the amateur singers felt increased joy and elation (Grape et al., 2002). The participants of my study noted similar outcomes in the ‘holistically good feeling’ (R22) or the ‘physical release’ (R120) they gained from singing. Correspondingly, some respondents felt the lockdown most acutely because it deprived them of these individual benefits. Rather than missing the ministry of chanting, one singer confessed missing the ‘personal singing experience’ (R76), while for another, the lockdown had made it impossible to ‘draw strength’ from singing (R111).

For many respondents, the singing experience, and the lack of it, extended from personal to collective. Analysing singing as an interplay between voicing and hearing others’ voices, ‘an embodied dialogue of inner and outer sounding and resounding’, Steven Feld (2003, p. 226) has suggested that collective voicing, as a physical act, heightens the interpersonal connection. As we saw in Table 1, the respondents generally acknowledged that singing enhanced their connection with other singers. In this light, it is understandable that one of the negative effects of the lockdown was deprivation of the collective physical singing experience. One respondent (R28) specifically mentioned that although she/he had had a chance to sing in a smaller group, singing *together with the choir* was lacking. Another described feeling even bodily discomfort for being denied the chance to sing together in person, at church:

I feel pains in my chest, and I am truly sad that I can’t go to church, even to listen. I have not been able to listen to the streamed concert by my own choir because I was so disappointed that I had not been able to sing at all during the lockdown. (R35)

#### Physical lockdown: new control and autonomy

Committed choral participation requires considerable effort in terms of travel and scheduling time to be physically present at rehearsals and divine service. The lockdown brought the weekly routines to a stop and released time. We noted earlier in the quantitative results that most of the respondents did not consider this as relieving, yet for some, the relaxation of busy schedules provided a new kind of control over their lives. In a very pragmatic manner, these respondents described how they needed to preserve their energy and how they valued the new chances to spend time, since attending worship physically was not possible. ‘My busy life has more time off, and no need to travel (= less haste and less schedules, rest)’, described one respondent (R85). Some regarded the lockdown conditions as stressful enough and thus saw the relaxing of church obligations as helpful: ‘Distance working has been exhausting. When the church has been closed, I have had energy for daily life’ (R117).

Their reactions can be associated with what Pargament et al. (2000) define as ‘self-directing religious coping’, that is, ‘seeking control through individual initiative rather than help from God’ (p. 523). By accepting the lockdown rationally, the respondents could focus on the challenges of the daily life and gain control in a situation in which the normal pace of life had radically changed.

The sense of control was intensified by the availability of live streamed services. As Metso et al. (2021) note in their survey-based analysis of the spiritual experiences among Orthodox and Lutheran Christians in Finland during the pandemic, the online transmissions empowered people to choose the circumstances in which they attended the service, which increased their autonomy. In my data, similar expressions of autonomy can be recognized. ‘Going to sing in the liturgy requires arrangements, so it is relieving when one can, with rather small organizing, attend the stream’, explained one respondent (R50). Another evaluated the rewarding aspects of being physically absent from church:

This is the first time in 20 years that I have free days during the weekend (Saturday and Sunday), when I don’t go to church. It has been a blessed experience, liberating, engendering silence. The family is happy when mother is at home for once. It is wonderful to listen to the [livestreams], no less blessed moments than being really at church. (R94)

The sacred space of the church entered the domestic space via TV screens and mobile devices. For some respondents, the virtual service loosened up certain ‘churchly’ behaviour, perhaps distancing the experience from the institutional forms of worship. As Luchenko (2021) points out, certain aspects of traditional churchgoing, such as a specific dress code, did not concern the digital participants; rather, they now had the control over how, when and where they attended worship. For example, the mobile device could extend the liturgical space outdoors:

Once I sat in a sunny spring forest and followed the service on the screen of my mobile phone. The birds took part in the singing! The old saying ‘Let the forest be my church’ [a line from *Paimenpoika* (A Shepherd Boy), a poem written by the Finnish poet Immi Hellén in 1898] got a new and a positive context. Of course, it was uncomfortable and thus a little comic . . . but beautiful. (R85)

#### Physicality of worship as comfort

Traditional Orthodox liturgy is founded on corporeal participation (Gschwandtner, 2019). While some respondents rejoiced over the chance to relax or do some chores while watching the live stream, there were also those who emphasized how they had continued to attend the online services as if physically present in the church, for example, by lighting candles in front of the icons, standing during the service, making the sign of the cross, bowing and prostrating: ‘When I listen at home, I sing along and observe all the customs in the service’ (R18). ‘I sing along at home with all the rituals’ (R22).

Keeping up these embodied liturgical customs at home, the respondents may have sought comfort in the continuity of worship as they knew it. For them, attending church, even if virtually, could still function as their coping strategy, enabling them to ‘engage in religious activities to shift focus from the stressor’ (Pargament et al., 2000, p. 523). By singing along and adhering to ‘all the customs’ of the physical liturgy, these respondents clearly wanted to demarcate the sacred in the domestic space and themselves as participators rather than mere ‘pious spectators’ (cf. Galadza, 2020).

Physical expressions of faith are common to Orthodox spirituality and their function can be seen as demarcating in normal times, as well. As Luehrmann (2018) describes,

the sacred act of praying is kept apart from everyday activities through its canonical, traditional forms and through the sensory manipulations that accompany it: lighting of candles, physical displacement to nearby or remote sacred places, shutting out external distractions through the use of a prayer book or icons. (p. 12)

Yet in the context of the live streamed services, the emphasis on ‘singing along with all the rituals’ may reflect the respondents’ need to mark their religious boundaries by sticking to the traditional physical practices of liturgy. According to Pargament et al. (2011), ‘clearly demarcating acceptable from unacceptable religious behaviour and remaining within religious boundaries’ (p. 56) can be seen as one way of seeking comfort in a crisis. In this sense, maintaining customs in a domestic setting also functions as an act of opposing the ‘unacceptable’, the disintegration of the collective ritual.

For many respondents, worship felt ‘real’ only in the church building. If we consider this from the perspective of coping, the difficulty to associate online services with real worship deprived the singers of the comfort of the religious focus: they could not find relief in attending church virtually, but, rather, the situation reminded them of what they were lacking (cf. Pargament et al., 2000). ‘When you are watching a livestream, praying does not work at all the same way as it does at church’, confessed one respondent (R121). The same was recognized by another: ‘Singing and praying give you strength and it is not the same thing to watch a stream at home. You have to be at church’ (R35). The church provided a frame for concentrating on prayer: ‘Although your eyes and mind might wander in the church, they always meet an icon or a *tuohus* [specific church candle made of beeswax] or a praying congregant. At home, there are so many things that distract your attention’ (R85). Participating in the worship simply ‘felt more’ when attended on site, in person (R81).

In an interesting way, these responses strongly emphasize the traditional practice of Orthodoxy as being focused on the communal liturgy in a sanctified space. Those who had access to church during the lockdown recognized the physicality of worship as comforting. ‘A physical place in the church feels good’, declared one respondent (R107). Yet at the same time, the experience of worship was inevitably recognized as lacking community. ‘It has been like a small feast to get to church, but at the same time the church has missed something: the people’, complained one respondent (R28). The absence of the congregation was sensed as physical silence, which felt as negative: ‘The silent church space exhausts all energy’ (R34).

#### The spiritual function of singing

Liturgical chanting constitutes a central medium of collective prayer in Orthodoxy. Through hymnody, the singers contribute to a sacred soundscape where the melodies amplify the spiritual content of the Orthodox hymn texts for the congregation to receive and participate in. Besides bearing the responsibility as performers of hymns, the singers have a prime opportunity to absorb the spiritual theological substance in hymnody because they have visual access to the hymn texts and they personally engage in verbalizing them.

The effect of singing on personal spiritual wellbeing was widely recognized by the respondents to the survey (see Table 1). Describing the spiritual quality of singing in general, the respondents highlighted gaining from it ‘a warm feeling of being close to God’ (R18), or ‘a great connection with everything’ (R111). For one respondent, singing was closely associated with praying and feeling connection with the church, ‘moreover, at church I feel closest to my departed close ones’ (R80). Another depicted singing as a profoundly transcendent experience, as ‘prayer and presence, in which I as a person move aside and give space to what sings in me’ (R101). Many others also emphasized the close connection between singing and prayer, explaining the integral role music has for their liturgical experience (cf. Kutarna, 2020). ‘Singing in services is pacifying and functions as praying’, explained one respondent (R64). It was this quality that distinguished sacred music from other recreation:

Singing in the choir is a natural part of my own spirituality – on one hand, it is a hobby that I like and consider positive, but on the other, it has a deeper meaning: it is thus an important part of my spiritual life, and this has to do with the idea of singing as a prayer. (R120)

For some, it was singing that brought about what they expected to feel in church in general:

[During singing,] I calm down and understand, for a moment, how I should live. I forget about useless cares. I feel closer to God, somehow on the right path. Perhaps I could get this feeling in church in any case, yet I have not been there many times without singing. Singing in a choir is generally great but singing in church is wonderful. (R44)

When the pandemic struck, this connection was severed for those singers who had no access to singing in worship. Some respondents revealed that the situation caused great anxiety. ‘Life lacks meaning a lot. You notice it when it’s missing’ (R69), stated one respondent. ‘What I have missed most is singing together and generally the divine services that nurture my soul’ (R52), described another, while for one respondent, the greatest longing was for the feeling of being close to God (R97). As we can estimate, attending the communal worship would traditionally have provided them with comfort and closeness to God, yet in the new situation, they had to find other ways.

#### Personal religious focus

Some respondents described how they had found a new kind of religious focus in life because of the lockdown. The crisis had disrupted old routines and forced them to rethink their personal spiritual state. The relaxing of busy schedules provided new possibilities to concentrate on the religious resources that were available, for example, on spiritual reading. Their previous familiarity with religious practices supported their positive religious coping (cf. Pargament et al., 2013).

I notice that the divine services have been significant for my life. Something has thus been missing [during the lockdown]. I have started to read the daily Scripture, according to the church calendar, as often as possible. Moreover, I began systematic reading of the Bible. There has been time to focus, without disturbance, on the spiritual life that I have grown into. (R86)

The sudden expansion of live streamed services to attend was also recognized as meaningful for the spiritual life.

Via streams, I have been able to ‘get to church’ even more often than normally, and, for example, I followed more Lenten services than usually. Moreover, the fact that I have just listened has, in a way, even deepened my spiritual life. (R64)

Thus, the deeper focus on personal spirituality, praying and spiritual matters (Pargament et al., 2000) served as a method of coping in the absence of communal worship and enriched the experience of these respondents.

Yet the lockdown created new obstacles to personal religious focus for those who were used to drawing spiritual strength from singing in worship. It is interesting that more than half of those respondents who had sung in the lockdown services perceived their task as stressful (see Table 2). This seemed to have to do with the quality expectations of which the performers were acutely aware. In the open responses, some reflected upon the anxious task of performing their own part without the support of other voices, which could be a real challenge for an unexperienced singer (R38; R81). Moreover, the fact that the live streams could be watched anywhere and often even at any time afterwards caused stress (R78). It is possible that the technical performance could shadow the experience of singing in liturgy and hinder the chance of focusing on its spiritual benefits. As one respondent described, singing in a live streamed service was far from a spiritual experience because of the possibility of being scrutinized by the audience:

All services are streamed to internet, so singing is really stressful! I know that a staggering number of people is following our parish [in social media] and watching our services, compared to the size of the parish. There’s a feeling that you have to sing well, or otherwise there is an audience at the other end that evaluates your successes and failures. It feels like you are in a concert or performing some show. The experience is empty and shallow. Singing in a service is like a show. (R19)

#### Disintegration of the singing community

Orthodox liturgy has a fundamentally communal character – both socially and theologically – which the pandemic challenged. For the church singers, it was not only the worship but also all the associated music activities, such as rehearsals and concerts, that were put on hold. Thus, apart from the physical benefits and the spiritual gratification gained from singing, they now lacked the social interaction, connection and inclusion which collective music making, especially choir singing, is known to provide (Daffern et al., 2021; Freeman, 2020; Moss et al., 2018).

The social dimension, that is, the sense of connection with the choir and the congregation, was recognized as an important aspect of singing in pre-pandemic conditions (see Table 1). The bonding achieved from choir singing was heightened by the sense of serving the whole community of worshippers. The qualitative responses manifest that the singers were generally highly conscious of their ministry as contributors to the collective experience of worship. ‘I sense joy and communality in singing. It is for me a kind of a task of obedience or “a volunteer work” in which I feel my small input brings good to the larger community’, described one respondent (R28). ‘I feel peace, even success, if I can take part in creating something beautiful for other congregants to listen to’, defined another (R12). Singing provided a strong sense of participating in worship, as the primary function for the congregation (R14; R27). The sense of ‘doing an important job’ was also recognized (R25; R98). Some perceived their own role in the wider context of the church. ‘I feel I am a mediator when I sing texts that are current to the church. I get satisfaction from being useful in this ministry’, said one respondent, while another quoted 1 Corinthians 12:12: ‘Communality is the most important feeling in singing at church. We form one body of Christ, in which all members of the church may participate. Singing in the choir is just one part of creating this feeling’ (R34). Some also emphasized the personal meaningfulness of singing together: ‘Singing has been an experience of belonging somewhere’ (R101).

#### Discontent as coping

Given the importance of communality, the disintegration of communities during lockdown was acutely witnessed in the responses. The reactions varied according to how the restrictions were adopted in each local parish: whether the cantor could invite singers to the services or not, and how these singers were selected. Different approaches to the lockdown caused clear discontent that was projected at the local church authorities or the cantor. ‘Why doesn’t anybody bother to organize [singing] here? [I am] disappointed!’ (R30) Another respondent complained the following: ‘I feel bitter. Why is there a quartet singing somewhere [else], while we only have the cantor?’ (R94) The selection of singers was also a cause of displeasure or envy for some. ‘It is unfair that certain people were invited to sing in the quartet during the lockdown’, complained one respondent (R103). Another respondent took a realistic stance at the situation yet could not deny negative feelings: ‘I understand why I can’t sing at church. Still, I am irritated that some are so privileged that they get to sing in a quartet’. (R35)

According to Pargament et al. (2000), feelings of ‘interpersonal religious discontent’ (p. 524), of dissatisfaction with the way the clergy or the members treat individuals in the stressful situation, may be seen as one way to cope with a critical situation. By taking the lockdown of singing personally, as cause for individual disappointment and bitterness, the singers may thus have looked for a way to relieve their anxiety.

We already noted that a majority of respondents did not consider the situation as unfair (see Table 3). Contrary to feeling envy, some appreciated the use of ‘competent singers’ at services (R34) or felt that the chosen singers ‘represented us all, for which I am grateful to them’ (R85). Nevertheless, more than half of the respondents without access to physical worship felt they had been excluded from their community (Table 3). ‘I have not been asked to sing at services and I feel excluded for it’, complained one respondent (R50). For some, the feeling went beyond simple disappointment, deep into the sense of meaningfulness of life in general. ‘My mind is as if broken. It feels miserable, not being able to meet fellow choir members or congregants’, described one respondent (R83). Although the reasons for the lockdown were understood, it was the abruptness of change that came as a shock, and the lack of communication made the exclusion palpable. One respondent (R35) concluded the following:

I have been upset for not having access to church, even as a regular congregant. The end of my only hobby has been upsetting and depressing. [. . .] I felt the church choir was the only place where I belonged, that I was one of the regulars there. Suddenly I wasn’t, and all contact was lost. It felt bad.

#### Providing comfort to others

The concern for communality also manifested in the responses of those who had sung in worship during the lockdown. Widely recognizing their own privilege in the situation (see Table 2), they seemed acutely aware of those who were left out. ‘I feel privileged for having the permission to be at church during worship, although I know there are a lot of people who would want to be there’, explained one respondent (R81). Interestingly, the joy for singing was mixed with a shade of guilt. ‘Because I had the chance to sing, I felt it was unfair towards those who had not been invited/asked’, said one singer (R107). Another singer suggested the following:

I have thought that singing is a privilege and a joy, yet at the same time I have felt a kind of sadness or guilt for being ‘chosen’. This might have perhaps been avoided, had the singing turns been rotated a little more equally. (R28)

Eventually, it was the sense of serving the whole community that helped respondents come to terms with the guilt. ‘I felt guilty especially at the start, when I knew how much the others would like to get to church. However, I perceived it as a ministry and did not stay and ruminate on it’, concluded one singer (R120). By seeing their role as representatives of a larger community, these individual singers could ease their conscience in a difficult situation. Ministry in this sense became extended into the virtual sphere, offering the singers a feeling of ‘providing spiritual support and comfort to others’ (Pargament et al., 2000, p. 524) by means of transmitted worship:

At times I feel guilty to go to church. There are many people for whom going to church is much more important than for me, and still I am invited to participate, just because of my singing hobby. I have tried to look at it as a ministry, because all services are streamed to internet, and maybe watching those videos has relieved somebody. (R78)

## Implications and limitations

Voicu et al. (2021) have showed how the pandemic, as a collectively experienced crisis, reinforced solidarity in some societies, while narrowed down social ties to family circles and close contacts in others. Within the smaller community represented in my data, the reactions seemed to vacillate between personal and collective: for some, the pandemic lockdown was an individual loss, while for others, it was predominantly a shared crisis to be overcome together.

Thus, the effects of the disrupted choral activities were manifested both in individual and collective experiences. The loss of the physiological benefits of singing and the bonding effects of group singing were acutely felt and might cause even bodily discomfort for some. The lockdown revealed the role of singing in worship for personal spiritual life, and those who could not participate generally saw the situation as spiritually challenging. However, some respondents who sang in the live streamed services questioned the spiritual reward they gained from their task due to the sense of performing to a scrutinizing audience. For many, being deprived of the chance to enjoy communality through singing caused sorrow and bitterness. Most notably, the lockdown disintegrated the worshipping community and created division in the choir, as some were allowed to sing, while others were not. This caused feelings of injustice and disappointment, and many felt excluded. Interestingly, and quite humanely, the privileged singers often felt guilty for being chosen to sing.

Explored within the framework of Kenneth Pargament’s and his colleagues’ religious coping theory, the results indicate that the church singers looked for different ways of processing the crisis. In an uncertain situation, many sought to maintain control over their life by engaging at a personal level in religious activities, such as praying and reading the Bible or spiritual books. In the absence of communal worship, some found that the personally maintained religious focus strengthened their spiritual life. Attending live streamed services at home as if physically present at church, with singing, candles, icons, prostrations and other gestures, could be seen as ways to gain comfort and to mark religious boundaries. By sticking to the liturgical routines, the singers could draw strength from the continuing connection with the church community as well as from demarcating the sacred from the loosening of the traditional worship behaviour, induced by the informality of observing live streamed services (cf. Luchenko, 2021).

At the same time, many intuitively welcomed a new kind of self-directing control over religious life provided by distance worship (Metso et al., 2021). The sense of autonomy was felt in many responses: liturgy could be attended and observed at the time and place most convenient for the individual. This undoubtedly poses a challenge for the future congregational life. The restoration of dispersed communities may not be entirely founded on the return to physical attendance but on calls for new ways to define the congregation and facilitate communal activities (Luchenko, 2021).

The pandemic had a disintegrating effect on communality and intimacy between people, which can be seen in the experience of exclusion among the respondents. From the perspective of coping, the expressions of discontent with the ways the restrictions were implemented in different parishes and the people responsible for it can be seen as a way to come to terms with the situation: ‘negative’ religious coping methods may also enhance wellbeing and are relevant for study (Pargament et al., 2011). In a similar way, the negative feelings of guilt expressed by some singers who were chosen to sing in the services could become channelled into attempts to provide spiritual support and comfort to others. It must be underlined that both positive and negative coping methods could be simultaneously traced in the responses of the same individual. For example, expressions of interpersonal discontent, such as disappointment at the implementation of the restrictions, could be accompanied by pragmatic attempts at controlling the crisis situation through individual initiative.

The author acknowledges several limitations of this study. An online questionnaire may not have been accessible to singers with little or no information and communication technology (ICT) skills (cf. Daffern et al., 2021). It is possible that digital illiteracy correlates with the difficulty to access live streamed worship that sustained at least some degree of connection with the church (cf. Metso & Ahonen, 2022). Thus, responses collected with a non-electronic questionnaire might have delved deeper into the experience of exclusion. During the pandemic, however, an e-survey appeared as a swift, convenient and the least risky medium. There is also a participant bias in the responses concerning the benefits of singing in worship because those who sing in worship are more likely to recognize such effects than those who do not (cf. Moss et al., 2018). In the quantitative section of the questionnaire, the questions were not standardized to ensure full comparability between the pre- and post-Covid perceptions of singing in worship, as their function was rather to lead to the qualitative, open-ended questions. The reliability of the responses to the scale investigating the reactions to (not) singing in worship during the lockdown was compromised by the fact that some participants responded to more than one set of questions, which could have been avoided by setting separate response paths within the questionnaire. In the 5-point Likert-type scale, the option ‘Cannot say’ was located between the affirmative and dissenting options, and some respondents may have considered it as equivalent of ‘neutral’. For the sake of clarity, these responses were excluded from the presentation of the results.

## Conclusion

The results of this mixed-methods study show that the Covid-19 lockdown in the spring of 2020 constituted a profound crisis for the amateur singers in the Orthodox Church of Finland and triggered different processes of coping among them. The results are consistent with earlier observations on the significance of singing as a physical act and especially of group singing as a source of bonding. They also highlight singing at worship as a spiritual action. Within the local choral communities, the pandemic forced individual singers to look for ways to gain control, comfort and intimacy in the absence of collective, physical worship. The individual experiences of church singers in the context of Orthodox Christian worship are a rarely examined topic. This has been the first study to empirically address the question of how Orthodox church singers coped during the pandemic that deprived them of the chance to sing together in worship. Future research could delve deeper into these experiences, for example, by using interview data that would enable more substantial and nuanced information about the psychological impact of the lockdown as integrated with the bodily, spiritual and communal dimensions of singing in worship. Moreover, a comparative analysis of experiences from other Christian denominations would open interesting perspectives to the roles of singers as well as the meaning of liturgical singing in different religious communities, in general.

## Notes

1. For comparison with Finland’s neighbours, in Sweden, the Greek Orthodox Church decided to suspend all public worship and celebrate online on 9 April (Heith-Stade, 2020), while in Russia, a similar announcement was made by the head of the Moscow diocese on 11 April (Luchenko, 2021).
